# Assessment of Expression LncRNA‐xloc‐000303, lncRNA‐LOC152578 and hsa‐miR‐29a in Patients Diagnosed With Colon Cancer in the Pre‐Treatment Stage

**DOI:** 10.1002/cnr2.70321

**Published:** 2025-09-15

**Authors:** Elham Tahami, Abolfazl Akbari, Reza Nekouian

**Affiliations:** ^1^ Department of Biotechnology Iran University of Medical Sciences Tehran Iran; ^2^ Colorectal Research Center Iran University of Medical Sciences Tehran Iran; ^3^ Pediatric Growth and Development Research Center, Institute of Endocrinology and Metabolism Iran University of Medical Sciences Tehran Iran; ^4^ Mehresoheila Cancer Charity Alborz Iran

**Keywords:** colorectal cancer, lncRNA‐LOC152578, LncRNA‐xloc‐000303, miR‐29a, pre‐treatment stage

## Abstract

**Background:**

Employing a panel of biomarkers may enable the early diagnosis of colorectal cancer. Among these biomarkers, long non‐coding RNAs (LncRNAs) and microRNAs (miRNAs) are significant due to their crucial roles in biological and metabolic processes.

**Aims:**

Therefore, the objective of this research was to evaluate the diagnostic value of the biomarkers LncRNA‐xloc‐000303, lncRNA‐LOC152578, and miR‐29a in the pre‐treatment stage of colorectal cancer in Iranian patients.

**Materials and Methods:**

In this study, 30 tumor tissue samples and 30 adjacent healthy tissue samples were collected, and RNA was extracted using the Trizol kit. Subsequently, the relative expression of the candidate microRNA and LncRNAs was analyzed using real‐time PCR. The obtained gene expression data were then analyzed using REST software.

**Results:**

In this study, it was found that the expression of LncRNA‐xloc‐000303 in tumor tissues significantly increased compared to healthy tissue (*p* < 0.001). Additionally, the analysis revealed that the expression level of lncRNA‐LOC152578 showed an increase in tumor tissues compared to healthy tissues (*p* = 0.57), although these changes were not statistically significant. The results of the hsa‐miR‐29a expression analysis indicated a down regulation in tumor tissues compared to healthy tissues; however, this change was also not statistically significant (*p* = 0.34).

**Conclusion:**

The results indicated that the expression changes of LncRNA‐xloc‐000303 in cancer tissue were significant compared to adjacent healthy tissue, suggesting its potential use as a biomarker for diagnosing colorectal cancer. However, further studies are needed to evaluate the biomarker specificity of lncRNA‐LOC152578 and hsa‐miR‐29a. This study was conducted in an Iranian population and its results can be used in the future for personalized medicine in the prevention and treatment of colorectal cancer.

## 1 Introduction

Colorectal cancer represents a significant public health challenge, ranking among the three most common cancers globally. In 2020, it represented 10% of all new cancer cases worldwide and was responsible for 9.4% of cancer‐related deaths globally [1]. This cancer is the second leading cause of cancer‐related deaths worldwide [2]. Timely prevention and treatment of colorectal cancer are crucial, as early diagnosis significantly enhances the likelihood of successful treatment [3]. The use of diagnostic biomarkers plays a pivotal role in the accurate and timely identification of the disease stage and the specific genes involved in its development [4]. The non‐invasive nature of biomarker‐based diagnostics makes them widely acceptable, contributing to improved treatment outcomes and survival rates for colorectal cancer patients [5].

Long non‐coding RNAs (LncRNAs), which have a role as a significant portion of non‐coding RNAs (ncRNAs), have minimal or no capacity for protein‐coding [6]. Nevertheless, they play a crucial role in various biological processes and cellular metabolisms [7]. LncRNAs are involved in a wide range of biological functions, consisting of translation, transcription, intracellular and extracellular transport, and the pathogenesis of various diseases. These molecules can interact with proteins, DNA, and RNA, participating in all levels of gene regulation, such as epigenetic modification, transcriptional control, and post‐transcriptional regulation [8, 9, 10]. LncRNAs have been shown to play a significant role in disease progression and are recognized as important biomarkers for the diagnosis and treatment of various conditions [11, 12].

MicroRNAs (miRNAs) are a kind of short non‐coding RNAs (ncRNAs), consisting of approximately 22 nucleotides, that repress protein translation by binding to target messenger RNAs (mRNAs). Bioinformatics and simulation studies suggest that miRNAs may regulate up to 30% of all human genes and hundreds of gene targets [13]. Increasing evidence indicates that miRNA expression performs a crucial function in different cellular processes involved in cancer [14], for instance cell growth, angiogenesis, differentiation, invasion, and epithelial‐mesenchymal transition [15]. Numerous studies have demonstrated that multiple miRNAs are aberrantly expressed in malignant cancers, including colorectal cancer [16, 17]. Functional research demonstrates that miRNAs can act as either tumor suppressors or oncogenes in human cancers [18], and they can influence various biological aspects of cancer, including chemoresistance [19] and metastasis [20]. Consequently, an increasing number of miRNAs that either promote or inhibit metastasis have been detected in tumor tissue expression profiles relative to non‐tumor tissues [21].

Despite the extensive studies conducted in this field, there is a need to test the hypotheses related to these studies in the Iranian population. The reason for this is that in genetic studies, the results of the study are greatly influenced by ethnic groups, both in terms of their genetic status and their environmental and contextual conditions. In the near future, personalized medicine will continue to expand, and this field requires raw information from different ethnic groups. Therefore, conducting this study in the Iranian population seems necessary.

In the near future, it is anticipated that utilizing a panel of molecular markers will enable the development of a distinct diagnostic kit to identify the stages of colorectal cancer, its metastasis or non‐metastasis, and the specific gene involved in its onset, thereby facilitating personalized treatment selection. This diagnostic approach aims to shield patients from the adverse effects of chemotherapy drugs that may not be universally suitable. Consequently, colorectal cancer patients can receive treatment that is cost‐effective, minimally invasive, and optimizes survival rates [22, 23]. In line with this objective, this study examined the expression of the molecular markers LncRNA‐xloc‐000303, lncRNA‐LOC152578, and hsa‐miR‐29a, assessing their diagnostic value in colorectal cancer during the pre‐treatment phase among subjects at the affiliated hospitals of Iran University of Medical Sciences. The practical goal of this study is to collect information on Iranian molecular markers and later place them in biobanks to be used for personalized medicine.

## 2 Material and Methods

### 2.1 Participants

The study population consisted of samples obtained from patients who underwent surgery at Jam and Firouzgar hospitals in Tehran, Iran. A total of 30 participants were included in this study. From each participant, both colon cancer tissue and surrounding healthy tissue were collected simultaneously. Following surgery and confirmation by a pathologist, the samples were placed in microtubes containing RNAlater and transported to the laboratory at 4°C for further processing. The study protocol was approved by the ethics committee of Iran University of Medical Sciences with registration number IR.IUMS.REC.1400.244. The patients gave informed consent.

### 2.2 Molecular Marker Selection

The three molecular markers were selected according to the literature and databases. Mir‐29a is known as a potential molecular marker and therapeutic target in colorectal cancer. However, this microRNA was not studied in Iran [24]. The LncRNAs LncRNA‐xloc‐000303 and lncRNA‐LOC152578 were introduced as the potential non‐coding RNAs [25].

### 2.3 RNA Extraction

In this study, RNA extraction from both tumor and healthy tissue samples was performed using the Trizol method. Tissues were homogenized and mixed with Trizol (Simbio, Iran), followed by the addition of chloroform, centrifugation to separate RNA, and precipitation with isopropanol. The RNA pellet was washed with ethanol, air‐dried, and rehydrated with RNase‐free water. The extracted RNA was stored at −70°C. RNA quantity and quality were evaluated using spectrophotometry and agarose gel electrophoresis, respectively.

### 2.4 cDNA Synthesis

Two different kits were used for synthesizing cDNA from LncRNA and microRNA molecules. To optimize cDNA synthesis from LncRNAs, a mixture of non‐specific Oligo‐dT primers and random hexamers was used with the Sambio cDNA synthesis kit (Simbio, Iran). For efficient cDNA synthesis from microRNAs, the VERNER kit (EcoTeb, Iran), along with specific Stem‐loop‐miR‐29a‐3p and Stem‐loop‐miR‐HK primers (listed in Table 1), and the MMLV enzyme were employed. The quality of the synthesized cDNA samples was evaluated using an RT‐PCR reaction targeting the β‐Actin gene, which served as an internal control (primer sequence provided in Table 2).

**TABLE 1:** Primer sequence of Stem‐loop‐miR‐HK and Stem‐loop‐miR‐29a‐3p used in cDNA synthesis.

Genes	Primer sequence (3′ → 5′)
Stem‐loop‐miR‐29a‐3p	GTCGTATCCAGTGCAGGGTCCGAGGTATTCGCACTGGATACGACACTCAC
Stem‐loop‐miR‐HK	GTCGTATCCAGTGCAGGGTCCGAGGTATTCGCACTGGATACGACATATTA

**TABLE 2:** Primer sequence of genes used in Real‐Time PCR.

Genes	Primers	Primer sequence (3′ → 5′)
β‐Actin	Forward	CGGAACCGCTCATTGCC
	Reverse	ACCCACACTGTGCCCATCTA
Lnc‐xloc‐000303	Forward	CCCTGTTGATTGACTTGTCTTG
	Reverse	CTTCTCTTGCTGTCTCCTACC
Lnc‐LOC152578	Forward	TTAAGCCAAGAAGTGAGG
	Reverse	ACGAAGGTGGTAACAGAG
miR‐29a‐3p	Forward	AACACGCAACATTCAACCTGT
	Reverse	GTCGTATCCAGTGCAGGGT

### 2.5 Real Time PCR

Primer sequences for β‐Actin, LncRNA‐xloc‐000303, lncRNA‐LOC152578, and hsa‐miR‐29a genes were initially designed using Beacon Designer software, and their specificity was confirmed using BLAST software (Table 2). The Real Time RT‐PCR reactions were carried out in a total volume of 15 μL, comprising 1 μL of cDNA, 1 μL of forward primer, 1 μL of reverse primer, 7.5 μL of Mastermix Cybergreen () 2X, and 4.5 μL of deionized water. The thermal cycling protocol commenced with an initial denaturation step at 95°C for 10 min, followed by 40 cycles. Each cycle included denaturation at 95°C for 15 s, annealing at 60°C for miRNA and 61°C for lncRNAs for 20 s, and extension at 72°C for 15 s. Following amplification, a melting curve analysis was performed by ramping from 55°C to 99°C to confirm the presence of specific PCR products based on their melting temperatures.

### 2.6 Data Analysis

To analyze the expression changes of the studied genes, data from Real Time PCR were processed using the Relative Expression Software Tool (REST). Additionally, GraphPad PRISM 10.3.1 software (GraphPad, US) was utilized to validate expression changes and analyze the quantitative expression levels of each gene, generating corresponding graphs. Statistical analyses were performed using SPSS 24 software (IBM Corp., US). For comparisons across three groups—Differentiated, TS, and Stage—Mann–Whitney tests (non‐parametric) and independent t‐tests (parametric) were employed. These analyses aimed to discern significant differences in gene expression among the various groups and stages of colorectal cancer.

## 3 Results

### 3.1 Population Characteristics

In this research, out of the 30 tumor samples and adjacent healthy tissues studied, 20 samples (66.66%) were obtained from male patients and 10 samples (33.33%) from female patients. Among the 30 cancer samples, seven were located in the colon (23.33%) and 23 in the rectum (76.66%). Among these, seven patients (23.33%) had polyps and 16 patients (53.33%) had colitis. Additionally, nine patients (30%) exhibited metastases. Clinicopathological analysis of the histological differentiation of the 30 collected samples revealed two cases of poor differentiation (6.66%), five cases of moderate differentiation (16.66%), and 23 cases of well differentiation (76.66%). Moreover, four samples (13.33%) indicated a genetic predisposition or family history of colon cancer. Statistical analysis of the cancer stage distribution among the samples showed that 19 samples (63.33%) were classified as stage II, eight samples (26.66%) as stage III, and three samples (10%) as stage IV.

### 3.2 Assessment of the Gene Expression

**FIGURE 1:**
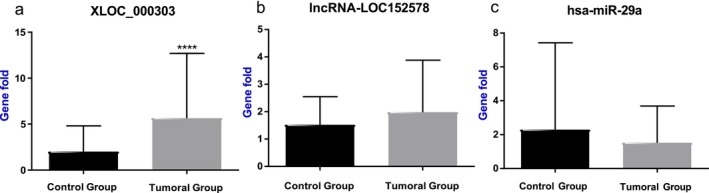
Expression changes of LncRNA‐xloc‐000303 (a), lncRNA‐LOC152578 (b), and hsa‐miR‐29a (c) genes in tumor tissues compared to healthy tissue with (****p < 0.0001).

The Real Time RT‐PCR analysis revealed significant upregulation of LncRNA‐xloc‐000303 expression in tumor tissues compared to healthy tissue (*p* < 0.0001) (Figure 1a). Conversely, while an increase in the expression of lncRNA‐LOC152578 (Figure 1b) and a decrease in the expression of hsa‐miR‐29a (Figure 1c) were observed in tumor tissues, these changes did not reach statistical significance.

### 3.3 Diagnostic Value of Biomarkers

**FIGURE 2:**
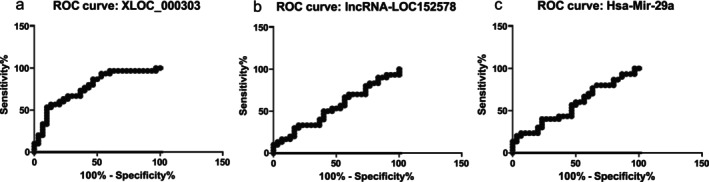
ROC diagram to evaluate the specificity and sensitivity of LncRNA‐xloc‐000303 (a), LOC152578 (b) and hsa‐miR‐29a (c) as colorectal biomarkers.

ROC statistical analysis was utilized to assess the diagnostic utility of markers in disease diagnosis. For LncRNA‐xloc‐000303, the analysis yielded an AUC of 0.779, a standard error of 0.06, a significant *p*‐value (= 0.0002), and an 89% confidence interval (CI) (Figure 2a). In contrast, the analysis of LncRNA‐LOC152578 indicated an AUC of 0.543, a standard error of 0.075, a non‐significant *p*‐value (< 0.564), and a 69% CI (Figure 2b). Evaluation of hsa‐miR‐29a showed an AUC of 0.571, a standard error of 0.074, a non‐significant *p*‐value (= 0.344), and a 71% CI (Figure 2c). These findings underscore the varying diagnostic potentials of these markers in colorectal cancer.

## 4 Discussion

Colorectal cancer ranks as the third most prevalent cancer and the second leading cause of cancer‐related mortality worldwide, with over 1.9 million new cases and 0.93 million deaths reported in 2020 [26]. The identification of molecular markers capable of predicting prognosis is a pivotal objective in cancer research. Increasing evidence underscores the active role of non‐coding RNAs, such as miRNAs and lncRNAs, in the regulation of cancer development and progression [27]. These RNA molecules also participate in controlling various biological processes, including tissue and organ growth, cell proliferation, apoptosis, and differentiation. The aberrant expression or dysregulation of miRNAs is implicated in tumor pathogenesis, progression, and resistance to therapies, making them a focus of extensive research efforts [28].

This study revealed that LncRNA‐xloc‐000303 exhibited significantly increased expression in colorectal cancer tissue compared to adjacent healthy tissue, suggesting its potential utility as a biomarker for future colorectal cancer prediction. The AUC of LncRNA‐xloc‐000303 was obtained as 0.779, which was in line with the literature that Shi et al. [25] reported the AUC of 0.919. However, this diagnostic accuracy was lower in the Iranian population. Conversely, although lncRNA‐LOC152578 also showed increased expression in tumor tissue, the change was not statistically significant. Due to limited existing research on LncRNA‐xloc‐000303 and lncRNA‐LOC152578, further investigations involving larger statistical populations are warranted for comprehensive evaluation. Regarding hsa‐miR‐29a, its expression was observed to decrease in colorectal cancer tissue compared to healthy tissue, yet this change was not significant. Numerous studies have reported conflicting findings regarding miR‐29a's role in cancer, suggesting it may act either to promote or suppress tumorigenesis depending on specific target genes. Therefore, additional research is essential to clarify the role of miR‐29a as a biomarker for colorectal cancer diagnosis and prognosis, as current evidence does not firmly support its predictive utility.

In a study on colorectal cancer cells (DLD1, SW620, and HCT116), the effects of aspirin treatment were investigated using microarray analysis. The study identified significant changes in the expression of 58 LncRNAs and 101 mRNAs in response to aspirin. Among the affected LncRNAs, 28 showed increased expression and 30 showed decreased expression upon aspirin treatment. Notably, LncRNAs NEAT1, LOC152578, GLYCAM1, and SARS exhibited significantly reduced expression levels following aspirin treatment compared to untreated colorectal cancer cells. These findings suggest that aspirin may impact specific LncRNAs involved in colorectal cancer, potentially influencing cancer‐related processes and treatment outcomes [25].

In 2018, Sun and colleagues investigated LncRNAs as potential biomarkers for predicting cervical squamous cell carcinoma. They found increased expression of four LncRNAs—HOTAIR, PVT1, XLOC‐000303, and AL592284.1—in cervical cancer compared to healthy samples, suggesting their potential as biomarkers for cancer prediction. Conversely, LOC152578, AJ420595, AC058791.2, and HULC showed no significant changes [29]. In the current study on colorectal cancer, LncRNA‐xloc‐000303 expression was examined compared to adjacent healthy tissue using Real Time RT‐PCR. The findings showed a significant increase (*p* < 0.0001) in LncRNA‐xloc‐000303 expression in tumor tissues, indicating its potential as a reliable biomarker for colorectal cancer prediction. Drawing on both studies, LncRNA‐xloc‐000303 emerges as a promising candidate for predicting cervical and colorectal cancers in future clinical applications. However, further research involving larger populations is essential to confirm these findings.

In recent studies, aspirin's impact on LncRNA expression in colorectal cancer cells has been explored. Chen et al. in 2021 found that aspirin treatment significantly altered the expression of over 10 000 LncRNAs and 22 000 mRNAs, with particular emphasis on how aspirin reduces LOC152578 expression, inhibiting tumor cell growth, and metastasis [30]. Similarly, Shi et al. [25] in 2015 observed changes in 58 LncRNAs and 101 mRNAs following aspirin treatment in colorectal cancer cells, with some LncRNAs such as NEAT1, LOC152578, GLYCAM1, and SARS showing reduced expression levels compared to untreated cancer cells. However, in the current study, the analysis of LncRNA‐LOC152578 expression in colorectal cancer tissues did not reveal significant changes compared to healthy tissue. This discrepancy highlights the need for further research, particularly larger studies, to better understand the roles of LncRNAs XLOC‐000303 and LOC152578 as potential biomarkers in colorectal cancer.

The present study examined the expression levels of XLOC‐000303, lncRNA‐LOC152578, and hsa‐miR‐29a in colorectal cancer cells compared to adjacent healthy tissue. While XLOC‐000303 and lncRNA‐LOC152578 showed increased expression in tumor tissues without statistical significance, hsa‐miR‐29a exhibited a non‐significant decrease in expression (*p* = 0.34). Previous research indicates that hsa‐miR‐29a may have dual roles in cancer biology, potentially promoting or suppressing tumor progression depending on specific target genes. Studies have highlighted its association with metastasis, drug resistance in breast cancer, and its anti‐tumor effects in gastric cancer, reflecting its complex role in different cancer types [31, 32]. Further investigations are necessary to elucidate its precise function in colorectal cancer and its potential utility as a biomarker [33, 34].

Brunet et al. [27] found increased miR‐18a and miR‐29a expression in stage III colorectal cancer patients compared to healthy individuals, suggesting their potential as diagnostic markers. Weissmann‐Brenner et al. [35] identified miR‐29a as a prognostic biomarker in stage II colorectal cancer, correlating its increased expression with disease progression and mortality. Huang et al. [36] demonstrated elevated miR‐29a levels in the serum of colorectal cancer patients compared to healthy controls, indicating its diagnostic potential. Wang et al. [37] observed higher miR‐29a expression in colorectal tumor tissues of liver metastatic patients compared to non‐metastatic cases, proposing its use in distinguishing metastatic cases. Overall, miR‐29a shows promise as a biomarker for cancer diagnosis, prognosis, and distinguishing between cancer subtypes, though further research is needed to fully validate its clinical utility. Yuan et al. [38] found that overexpression of miR‐29a increases the risk of colorectal cancer recurrence. Their study also indicated that miR‐29a plays a role in modulating drug sensitivity in colorectal cancer cells. Tang et al. [33] showed that downregulation of miR‐29a expression significantly reduces the malignant characteristics of colorectal cancer cells. Due to conflicting results from various studies, further investigation is essential to clarify the role of miR‐29a as a biomarker for prognosis in cancer patients, particularly in colorectal cancer. Current evidence does not provide conclusive support for its use as a prognostic indicator, highlighting the need for additional research to establish its clinical significance.

Regarding LncRNA‐xloc‐000303, although statistical significance was achieved, it should be noted that statistical significance is different from biological significance. To prove biological significance, there needs to be strong support from the scientific literature so that it can be said that there is biological plausibility. For this purpose, bioinformatics studies can be helpful.

Regarding the future perspective of this study, some molecular markers can be affected by therapeutic interventions. For example, vitamin D can alter the levels of these markers. In addition, it should be said that personalized medicine will expand in the near future [39]. In personalized medicine, biomarkers, including microRNAs, will be of great importance in the diagnosis, prognosis, and treatment of diseases. Colon cancer is no exception and, given its burden on public health in the Iranian population, requires special attention. The results of this study are based on the genetic and environmental status of the Iranian population and, as a result, can contribute to the personalized medicine approach in Iran. In addition, repeating such studies in different societies can show the generalizability of the results, and these repeated studies can later be used in systematic review studies.

The strength of this study is that the present study is the first pilot study for such ncRNAs panel in colorectal cancer in Iran. Despite its strengths, our study had some limitations. Among these limitations was the small sample size, which limits the generalizability of the study results. However, our statistical power was sufficient to detect some significant relationships. Another limitation was the lack of molecular markers in the blood. Although it seems that the histological examination of these markers is a bit late, it should be borne in mind that biopsies are also performed in the early stages of cancer and also from precancerous lesions. Therefore, histological examination also has its value. One of the limitations of this study was the lack of bioinformatics analysis of microRNAs and LncRNAs. These studies can be a source of hypothesis generation for laboratory and clinical studies. It is suggested that these studies be performed in future studies. As well, for future studies, conducting this research on a larger sample and in different clinical stages and tumor types is suggested. Also, using animal and cell models is suggested for further validation.

## 5 Conclusion

Given the limited sample size and statistical population in this study, extending the research with increased sample size and duration is recommended to enhance result robustness. Furthermore, investigating other demographic groups would strengthen the generalizability of findings. Additionally, due to the insufficient comparative data on biomarkers LncRNA‐xloc‐000303 and lncRNA‐LOC152578, it is advisable to evaluate their expression in larger and diverse populations, including other types of cancers. Moreover, designing and implementing experiments using cell and animal models would facilitate elucidation of the biological roles of LncRNA‐xloc‐000303. The results of this study highlight the potential of LncRNA‐xloc‐000303, lncRNA‐LOC152578, and hsa‐miR‐29a as valuable biomarkers for the early detection and management of colon cancer. Their differential expression in patients suggests they could aid in diagnosis and possibly guide treatment decisions. With further validation, these biomarkers may contribute to more precise and personalized approaches in clinical oncology.

## Author Contributions

**Elham Tahami:** data collection, drafting. **Abolfazl Akbari:** design and conceptualization, critical revision. **Reza Nekouian:** design and conceptualization, critical revision. All the authors approved the final version of the manuscript.

## Conflicts of Interest

The authors declare no conflicts of interest.

## Data Availability

The data that support the findings of this study are available from the corresponding author upon reasonable request.
